# Breast Dense Tissue Segmentation with Noisy Labels: A Hybrid Threshold-Based and Mask-Based Approach

**DOI:** 10.3390/diagnostics12081822

**Published:** 2022-07-28

**Authors:** Andrés Larroza, Francisco Javier Pérez-Benito, Juan-Carlos Perez-Cortes, Marta Román, Marina Pollán, Beatriz Pérez-Gómez, Dolores Salas-Trejo, María Casals, Rafael Llobet

**Affiliations:** 1 Instituto Tecnológico de la Informática, Universitat Politècnica de València, Camino de Vera, 46022 València, Spain; fjperez@iti.es (F.J.P.-B.); jcperez@iti.upv.es (J.-C.P.-C.); rllobet@iti.upv.es (R.L.); 2 Department of Epidemiology and Evaluation, IMIM (Hospital del Mar Medical Research Institute), Passeig Marítim 25–29, 08003 Barcelona, Spain; mroman@parcdesalutmar.cat; 3 National Center for Epidemiology, Carlos III Institute of Health, Monforte de Lemos, 5, 28029 Madrid, Spain; mpollan@isciii.es (M.P.); bperez@isciii.es (B.P.-G.); 4 Consortium for Biomedical Research in Epidemiology and Public Health (CIBER en Epidemiología y Salud Pública—CIBERESP), Carlos III Institute of Health, Monforte de Lemos, 5, 28029 Madrid, Spain; 5 Valencian Breast Cancer Screening Program, General Directorate of Public Health, 46022 València, Spain; salas_dol@gva.es (D.S.-T.); casals_mar@gva.es (M.C.); 6 Centro Superior de Investigación en Salud Pública, CSISP, FISABIO, 46020 València, Spain

**Keywords:** mammography, breast density segmentation, deep learning, noisy labels

## Abstract

Breast density assessed from digital mammograms is a known biomarker related to a higher risk of developing breast cancer. Supervised learning algorithms have been implemented to determine this. However, the performance of these algorithms depends on the quality of the ground-truth information, which expert readers usually provide. These expert labels are noisy approximations to the ground truth, as there is both intra- and inter-observer variability among them. Thus, it is crucial to provide a reliable method to measure breast density from mammograms. This paper presents a fully automated method based on deep learning to estimate breast density, including breast detection, pectoral muscle exclusion, and dense tissue segmentation. We propose a novel confusion matrix (CM)—YNet model for the segmentation step. This architecture includes networks to model each radiologist’s noisy label and gives the estimated ground-truth segmentation as well as two parameters that allow interaction with a threshold-based labeling tool. A multi-center study involving 1785 women whose “for presentation” mammograms were obtained from 11 different medical facilities was performed. A total of 2496 mammograms were used as the training corpus, and 844 formed the testing corpus. Additionally, we included a totally independent dataset from a different center, composed of 381 women with one image per patient. Each mammogram was labeled independently by two expert radiologists using a threshold-based tool. The implemented CM-Ynet model achieved the highest DICE score averaged over both test datasets (0.82±0.14) when compared to the closest dense-tissue segmentation assessment from both radiologists. The level of concordance between the two radiologists showed a DICE score of 0.76±0.17. An automatic breast density estimator based on deep learning exhibited higher performance when compared with two experienced radiologists. This suggests that modeling each radiologist’s label allows for better estimation of the unknown ground-truth segmentation. The advantage of the proposed model is that it also provides the threshold parameters that enable user interaction with a threshold-based tool.

## 1. Introduction

Mammogram screening is a highly standardized method used in breast cancer early detection programs, with obtained mammograms examined in batches of up to 50 per hour by qualified radiologists [1]. Full field digital mammography (FFDM) is one of the most used screening methods for breast cancer. Improved imaging features are now available due to technological advancements, allowing early diagnosis of breast cancer.

Percent density (PD), which assesses the proportion of fibroglandular tissue in the whole breast, is recognized to be a risk factor for breast cancer [2,3]. The American College of Radiology Breast Imaging Reporting and Data System (BI-RADS) has published a breast classification system based on density, shape, granularity of dense tissue, and probability of masking [4]. The classification criteria imply that not only the overall amount of dense tissue, but also its distribution and ability to hide lessions, is relevant [5,6]. Furthermore, inter- and intra-observer variability is one of the major issues in PD evaluation [7,8,9,10].

The development of an automated technique that has a high degree of concordance with several radiologists might be one of the first steps towards standardizing breast density readings. The authors of [11] point out that an automated tool may be used as an independent second reader of screening mammograms when double reading is required. A second human reader would only be needed to resolve differences between the first human reader and the system, reducing the effort for any screening program that uses double reading.

Convolutional neural networks (CNNs) are one of the most used paradigms in computer vision issues handled using deep learning [12]. They are based on the extraction of higher-order characteristics as the images pass through successive layers. For many recognition and detection tasks, CNNs are currently the state-of-the-art [13,14,15].

In a previous study [16], we introduced a fully automated framework for dense-tissue segmentation. It included breast detection, pectoral muscle exclusion, and dense-tissue segmentation. The dense-tissue segmentation step was implemented as a regression deep learning architecture, named the entirely convolutional neural network (ECNN), whose output was the two parameters used as intrinsic segmentation features.

In the present investigation, we explore further the dense-tissue segmentation step by implementing a new architecture, which we named “confusion matrices-YNet” (CM-YNet) as it is based on the state-of-the-art technology [17,18] for medical image segmentation when the ground-truth label is unknown. This model learns the characteristics of the individual expert annotators (e.g., tendency to over- or under-segment) by estimating the pixel-wise confusion matrices on a per-image basis, and outputs the estimated pixel-wise segmentation mask and the two aforementioned segmentation parameters for threshold-based segmentation. The availability of the segmentation parameters is essential to retain compatibility with threshold-based tools so that users can easily edit inaccurate segmentations.

Among the contributions of this work, we can highlight: (1) a new segmentation architecture CM-YNet that generates an estimation of the ground-truth dense-tissue segmentation mask and two parameters for compatibility with threshold-based segmentation tools; (2) the proposed model outperforms other deep learning architectures when compared on the same dataset; (3) a loss function that jointly learns the segmentation mask, threshold parameters; and, finally, (4) the ability to manually modify the segmentation using threshold-based software such as DMScan [19,20].

## 2. Materials and Methods

### 2.1. Datasets

A multi-center study covered women from 11 medical centers of the *Generalitat Valenciana* (GVA) as part of the Spanish breast cancer screening network. It included 1785 women with ages from 45 to 70. The cranio-caudal (CC) and medio lateral-oblique (MLO) views were available for 10 out of 11 of the centers, while one center only collected the CC view. This dataset was used for training, validation, and testing. The dataset was randomly partitioned into 75% (2496 mammograms) for training and validation (10%), and 25% for testing (844 mammograms). The mammograms of the same patient were always included in the same set.

Additionally, an independent dataset composed of 381 images obtained at the *Institut Hospital del Mar d’Investigacions Mèdiques* (IMIM) was included only for testing to obtain a better evaluation of the generalization performance of the models. Because the researchers at IMIM had a particular interest in testing the fully automated tool in various types of images, 283 out of the 381 images at IMIM were obtained from old acquisition devices with lower image quality, making the segmentation task more challenging. Only CC views were provided for this dataset.

Since in Spain “raw” mammograms are not routinely stored, all the mammograms are of the type “for presentation”. All mammograms were segmented independently by two expert radiologists (referred to in this paper as R1 and R2) using DMScan [19,20].

A summary of the data used in this study is presented in Table 1.

### 2.2. Segmentation Pipeline

**Figure 1:**
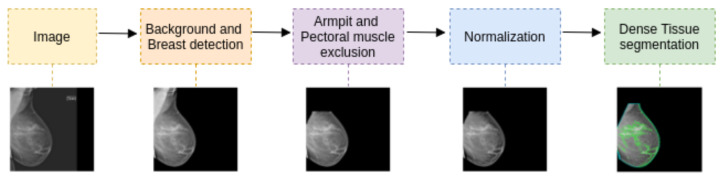
Segmentation pipeline. The last step is the deep-learning-based model for dense-tissue segmentation, which is the focus of the present investigation.

The segmentation pipeline consists of the steps summarized in Figure 1: a first step covering breast detection and pectoral muscle exclusion, a second step to exclude armpit and pectoral muscle, a third step to normalize the histogram variability between acquisition devices, and finally, a deep learning model carrying out the dense-tissue segmentation task. The focus of the present work is the dense-tissue segmentation step as we compare different deep learning-based approaches.

#### 2.2.1. Background and Breast Detection

An iterative algorithm based on connected components obtains the gray level threshold that distinguishes the breast from the background [21]. Based on the premise that the most frequent pixel value has to belong to the background, a range of possible breast thresholds is determined by taking all the unique values in the image. Then, this range of thresholds is covered until only two homogeneous components are detected. The breast must be left-oriented, and the image is binarized using the first possible threshold before the connected component labeling algorithm, named scan-plus-array-based union-find (SAUF), is applied. Finally, if only two components are obtained, the algorithm ends, otherwise, the range of possible thresholds is continuously covered.

#### 2.2.2. Armpit and Pectoral Muscle Exclusion

Assuming that the pectoral muscle appears in a triangle in one of the top corners of the image, an algorithm based on negative gradient changes is implemented. The algorithm requires the breast image to be left-oriented. A Gaussian filter with σ=3 and a 50-pixel moving window is then applied to smooth edges and remove isolated bright pixels. We empirically chose the optimal values after several trials as these parameters depend on the image resolution and object size. As the muscle border is well-defined, it tends to be the last remaining part after the smoothing process. We iteratively built a polygon that encloses the exclusion area by selecting the pixel with the lowest gradient every 50 rows until the column of the selected pixel was close enough to the left image border. Finally, the vertex that closed the polygon was taken to be the first pixel from the top left corner.

#### 2.2.3. Histogram Normalization

The different mammogram acquisition devices show huge variability in the quality of mammograms. As demonstrated previously in [16], this variability was found to be significant and negatively impacted the training of a machine learning model. Therefore, normalization among acquisition devices was performed by applying the following steps:

1. Normalize the pixel values of the image between [0,1].
2. Shift the histogram to set the minimum breast tissue pixel to 0.
3. Normalize the pixel values again between [0,1].
4. Standardize the breast pixel values to a normal distribution *Z*∼N(0,1).
5. Adjust the pixel values so that the mode is 0.
6. Assuming that most typical percent density values are below 30% (above the 70th percentile), and values under the 30th percentile only belong to fatty tissue, apply a linear stretching from percentile 30 to −1 and from percentile 70 to 1.
7. Apply oa normalization once more to ensure the inputs for the deep neural network are between [0,1].

### 2.3. Dense Tissue Segmentation

Dense tissue segmentation can be obtained using two different approaches: (a) parametric approximation, in which threshold parameters are applied to the image to obtain the segmentation mask; and (b) mask approximation, which consists of directly assigning each pixel in the image as dense tissue or not. The advantage of using the mask approximation is that it can be used for any kind of segmentation as it is estimated at pixel level, but the expert labels should be defined by manually contouring the region of interest (ROI). Therefore, the parametric approximation is more convenient for manual interaction with the expert as only a few (usually two or three) parameters need to be adjusted.

In a previous approach [16], the entirely convolutional neural network (ECNN) architecture was presented for dense-tissue segmentation. The DICE scores obtained with ECNN were close to the concordance achieved between the two radiologists. However, the performance on images with low gray-level resolution was not optimal. Therefore, in this study, we explore other architectures that directly estimate the segmentation mask by modeling each annotator’s label independently. Finally, a new architecture named ”confusion matrices-YNet“ (CM-YNet) is proposed, which aims to estimate the dense-tissue mask and the two segmentation parameters simultaneously.

#### 2.3.1. ECNN: Parameter Estimation

The ECNN model estimates two segmentation parameters which are learned as image-level features. The estimated parameters, the brightness corrector α and the fibrograndular tissue threshold $th_{F}$, are then used to replicate the segmentation provided by DMScan [19,20], the tool used to generate the labels by the expert radiologists.

#### 2.3.2. U-Net: Mask Estimation

To directly obtain the dense-tissue mask, we first experimented with the well-known segmentation network U-Net [22]. U-Net is composed of an encoding network and a decoding network. The encoding network consists of a stack of down-sampling blocks of double convolutions, while the decoding network consists of up-sampling blocks. Skip-connections between the encoder and the decoder allow information to be shared between them.

Our implementation uses three down-sampling stages with 24, 48, and 96 channels for each encoder. We applied instance normalization [23] to each encoder and decoder layer.

#### 2.3.3. Y-Net: Hybrid Approach

The Y-Net model proposed by Mehta et al. [18] jointly performs segmentation and classification of different types of tissues in breast biopsy images. The U-Net architecture was generalized by adding a parallel branch that outputs a classification label. The results demonstrated that the joint learning implemented within Y-Net improved diagnostic accuracy. Our implementation of Y-Net follows the same idea, but the parallel branch predicts the segmentation parameters α and $th_{F}$. Therefore, with the Y-Net model, we can simultaneously estimate the dense-tissue mask and the segmentation parameters that would allow manual modifications with a threshold-based tool.

**Figure 2:**
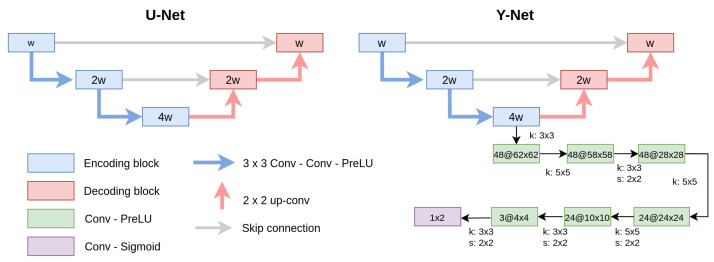
U-Net and Y-Net architectures. Y-Net jointly estimates the dense-tissue mask and the segmentation parameters (α and $th_{F}$) needed to segment the dense tissue with a threshold-based tool. The channel width (w), kernel (k) and the stride (s) size for each layer are shown for the parallel branch incorporated in Y-Net.

The parameter estimation branch consists of several convolution layers added from the last encoder layer of the U-Net. Similarly, as in ECNN, the convolutions reduce the inputs until the segmentation parameters are extracted. Figure 2 shows a general diagram of the U-Net and Y-Net architectures.

#### 2.3.4. CM-Segmentation: Learning Noisy Labels

The lack of a unique ground truth due to inter-reader variability [11] means that it is normally necessary to train the deep learning models using the segmentation of both radiologists as independent annotations, or by fusioning both labels. Either approach can yield a high degree of concordance between the model and the annotators. However, the available expert labels are noisy approximations of the unknown ground-truth segmentation mask. To tackle this phenomenon, Zhang et al. [17] proposed an architecture consisting of two coupled convolutional neural networks (CNNs):

1. The first is the segmentation network that estimates the true segmentation.
2. The second is the annotation network that models the characteristics of individual experts by estimating the pixel-wise confusion matrices (CM).

The annotation network shares the same parameters as the segmentation network apart from the last layers. It estimates the CMs at each spatial location, thus yielding a c×c output, where *c* is the number of channels, which is two for a binary segmentation, as in our case. We carried out experiments incorporating the annotation network of different segmentation networks: CM-ECNN, CM-UNet, and the proposed CM-YNet.

#### 2.3.5. CM-YNet: Proposed Network

**Figure 3:**
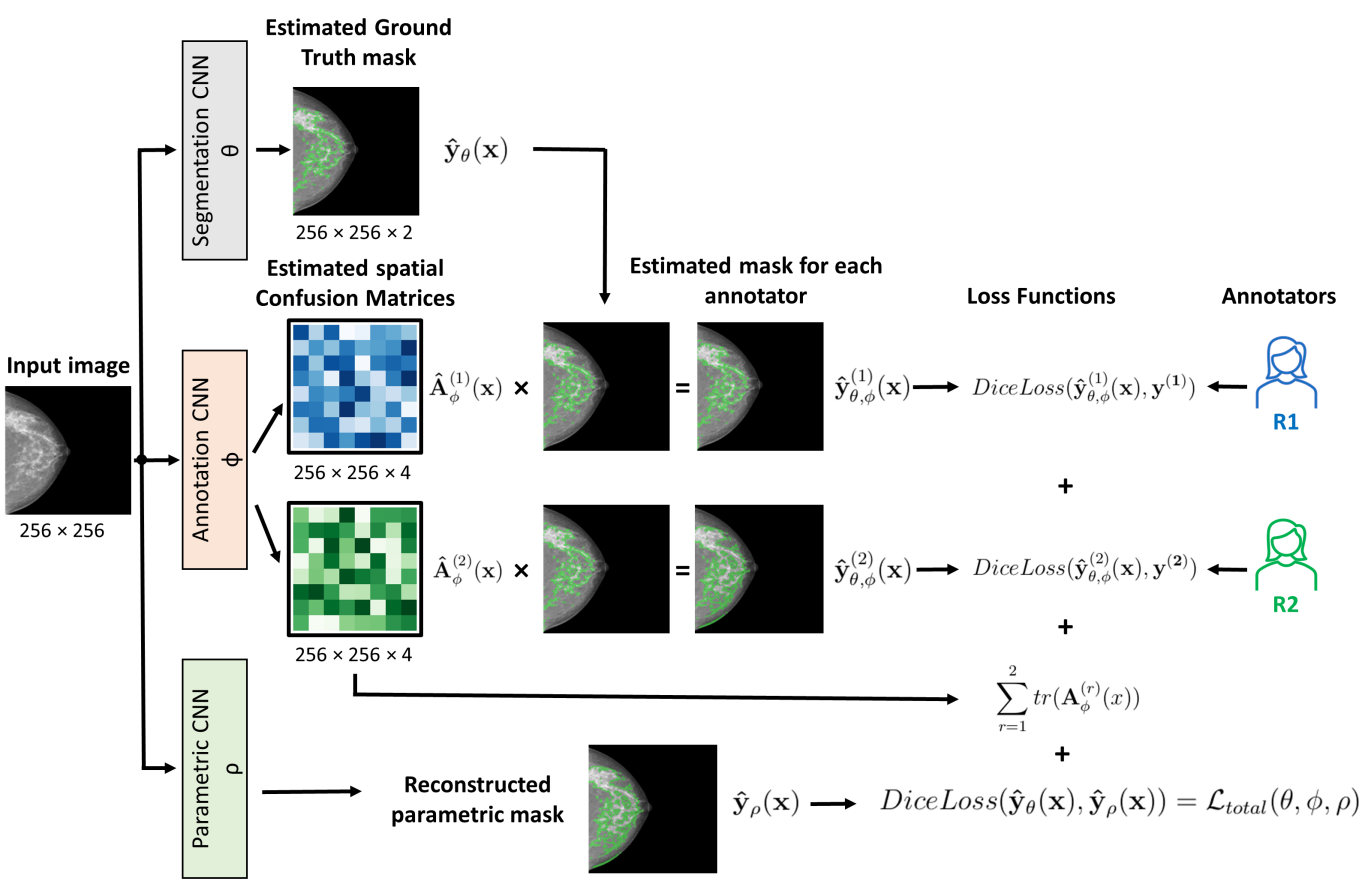
Schematic representation of the proposed (CM-YNet) architecture showing how the total loss function is computed. The segmentation and parametric convolutional neural networks (CNNs) are integrated in the Y-Net model described above. At inference, the output of the segmentation network ${\hat{y}}_{\theta }\left(x\right)$ is used to obtain a new prediction.

Based on the networks described above, we propose a CM-YNet architecture which models each radiologist’s label to estimate the dense-tissue mask and segmentation parameters for compatibility with threshold-based tools. The structure of the proposed CM-YNet model is shown in Figure 3.

The loss function to jointly learn the segmentation mask, segmentation parameters, and confusion matrices for each annotator is implemented as follows:

(1) $$L_{total}\left(\theta ,\phi ,\rho \right)=L_{\left(\theta \right)}+\alpha .L_{\left(\phi \right)}+\beta .L_{(\rho ),}$$

where the segmentation, annotator, and parameter networks are represented by θ, ϕ, and ρ, respectively. The parameter α is the weight of the annotation network loss, while β is the weight of the parametric loss.

The first term of the total loss function is given by Equation (2):

(2) $$L_{\left(\theta \right)}=\sum_{r=1}^{n}DiceLoss\left({\hat{y}}_{\theta ,\phi }^{\left(r\right)}\left(x\right),y^{\left(r\right)}\right),$$

where ${\hat{y}}_{\theta ,\phi }^{\left(r\right)}\left(x\right)$ represents the estimated segmentation probability map of the corresponding annotator, obtained by the element-wise matrix multiplication of the output of the segmentation network ${\hat{y}}_{\theta }\left(x\right)$ and its corresponding confusion matrix, while $y^{\left(r\right)}$ is the label of the annotator *r*. Minimizing this term encourages each annotator-specific prediction to be as close as possible to the true noisy label distribution. However, this loss function alone is not capable of separating the annotation noise from the true label distribution. There are many possible combinations of the CM and the segmentation model that perfectly match the true annotator’s distribution for any input x. To deal with this problem, the trace of the estimated CMs is added according to Equation (3):

(3) $$L_{\left(\phi \right)}=\sum_{r=1}^{n}tr\left(A_{\phi }^{\left(r\right)}\left(x\right)\right),$$

where $A_{\phi }^{\left(r\right)}\left(x\right)$ is the spatial CM for annotator *r*. Minimizing the trace encourages the estimated annotators to have maximum unreliability, thus acting as a regularization term.

Finally, the last term of the total loss function is given by Equation (4):

(4) $$L_{\left(\rho \right)}=DiceLoss\left({\hat{y}}_{\theta }\left(x\right),{\hat{y}}_{\rho }\left(x\right)\right),$$

which corresponds to the parametric loss, namely, the DICE loss between the predicted ground truth ${\hat{y}}_{\theta }\left(x\right)$ and the mask reconstructed with the predicted segmentation parameters ${\hat{y}}_{\rho }\left(x\right)$. Minimizing this function ensures that the reconstructed mask is as close as possible to the ground-truth mask estimated by the segmentation network θ.

### 2.4. Implementation Details

The algorithms were implemented in Pytorch and trained for a maximum of 500 epochs. The epoch with the lowest validation loss was saved and used for test predictions. All the models were trained on an NVIDIA Tesla V100 using a batch size of eight, a learning rate of 0.0001, and the AdamW [24] optimizer with default parameters.

The Dicom input images were resized to 256×256 pixels. Data augmentation was performed during training with random vertical flips assuming that all the images were left-oriented, as described in Section 2.2.2.

We trained each of the described models (ECNN, U-Net, and Y-Net) in three different ways:

- Using the segmentation of both radiologists as independent annotations. Each image is seen twice during training with this approach, thus doubling the training corpus.
- Using the AND mask as the ground-truth label, obtained as the pixel-wise intersection of both annotations.
- Using the CM approach described in Section 2.3.4.

We performed several experiments to search the optimal values for the parameters α and β of the total loss function (Equation (1)). Both parameters were searched within the values [0.1,0.3,0.5,0.9], and those that yielded the best results in the validation data were selected: α=0.9 and β=0.1. The annotation network in the CM-based models was pre-trained for 10 epochs as a warm-up to maximize the trace of the CMs to encourage diagonal dominance, as suggested in [17].

### 2.5. Evaluation

To measure the performance of the models, we chose the widely used Sørensen–Dice similarity coefficient [25] which measures how much two masks $M_{1}$ and $M_{2}$ overlap, according to Equation (5).

(5) $$DICE\left(M_{1},M_{2}\right)=\frac{2|M_{1}\cap M_{2}|}{|M_{1}\left|+\right|M_{2}|}$$

Our experiments were based on the assumption that the ground-truth (GT) label is not known. Therefore, we compared the results against each expert annotation independently and also obtained the mean DICE score between the estimated mask and the expert label which was closest to it. As we only had the labels of two experts available, the only label fusion implemented for evaluation was the pixel-wise logical-AND between the binary masks generated by the two radiologists.

The DICE scores obtained by the different models were compared using a one-way ANOVA and Tukey’s range test statistic. The *p*-values were considered statistically significant at the 0.05 cut-off. The Pearson correlation coefficient was used to compare breast percent density (PD) as measured by our method and each radiologist’s assessment.

## 3. Results

### 3.1. Comparison of Different Models

The DICE scores for the different models are presented in Table 2 for the GVA and IMIM datasets. The mask approximation models (U-Net and YNET-mask) achieved better performance (p<0.001) than their parametric counterparts (ECNN, YNet-param) for all training variations. The parametric output of CM-Ynet did not achieve the performance of the mask output. However, this was not so relevant as the parametric output was only intended to maintain compatibility with the threshold-based tool when some user interaction is needed. CM-Ynet (param.) outperformed the previously implemented ECNN in this regard. Even though the expert labels used to train the model were obtained by setting the parameters predicted by the parametric models, these only have two degrees of freedom, and, therefore, the generalization performance could not outperform the mask approximation models, which have the freedom to adjust all pixels individually.

**Figure 4:**
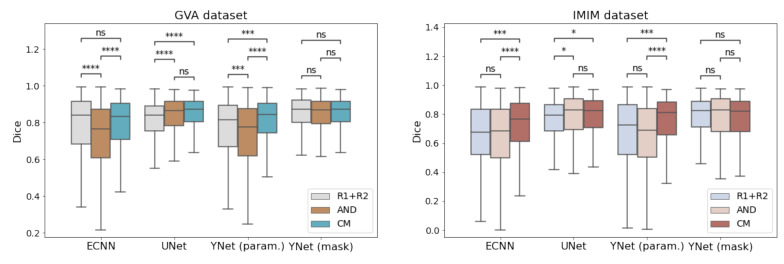
Comparison of models trained with different strategies (R1 + R2, AND mask and confusion matrices (CM)). ns: not significant, *: p≤0.05, ***: p≤0.001, ****: p≤0.0001.

**Figure 5:**
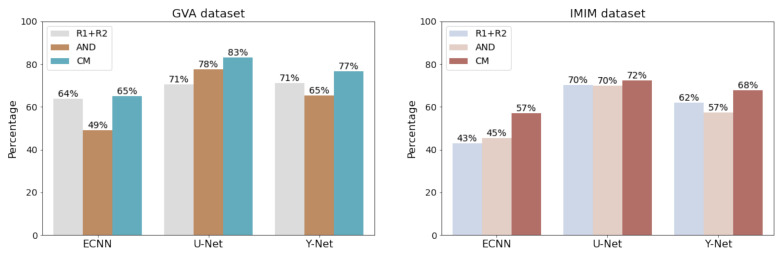
Percent success for each model. A sample in the test set is counted as successful if the Dice score between the estimated mask and the closest radiologist is higher than the concordance between the radiologists (0.77 for GVA and 0.72 for IMIM). The Y-Net includes the parametric and mask outputs.

We should also note that the implementation of the CM versions (CM-ECNN, CM-UNet, and CM-YNet (param.)) outperformed those trained using each radiologist’s label as independent ground truth and those trained with the AND masks (Figure 4). The difference between CM-YNet (mask) with AND-YNet (mask) and YNet (mask) was not statistically significant. However, the advantage of training the Y-Net model using the confusion matrices approach is still supported based on the following:

1. The CM versions for ECNN, U-Net and Y-Net were more stable as they showed lower errors.
2. Including more images for training with labels from different annotators is straightforward due to the network configuration. This functionality allows for easy improvement of the model generalization by adding images from other centers in “for presentation” or “for processing” formats.
3. The overall performance (parametric and mask outputs) was better than for the other methods as shown in Figure 5.

### 3.2. Comparison per Acquisition Device

The results per mammography facility are shown in Table 3. The ECNN model only achieved better performance in mammography facility 07. Notably, the agreement between radiologists was the highest for this acquisition center. The mask approximation yielded higher scores in all other centers. Centers 04, 18, and 22 corresponded to devices with low gray-level resolution. In these cases, the improvement with CM-YNet over ECNN was much higher.

These results demonstrate a good level of concordance of CM-YNet with the segmentation provided by experienced radiologists. As can be seen in Table 1, the mammography facilities with a FUJIFILM device (centers 01, 02, 05, 11, 13 and 21) were those that presented better results for ECNN, while performance dropped significantly on most of the other devices. In contrast, CM-YNet (mask) performed similarly on all devices (DICE>0.8 with the exception of center 22 whose images were of very low quality. It is noteworthy that images from centers 21 and 22 were never seen during training as these corresponded to the IMIM dataset that was used only for testing. CM-YNet (mask) also outperformed both ECNN and the radiologists in all cases except for center 07.

**Figure 6:**
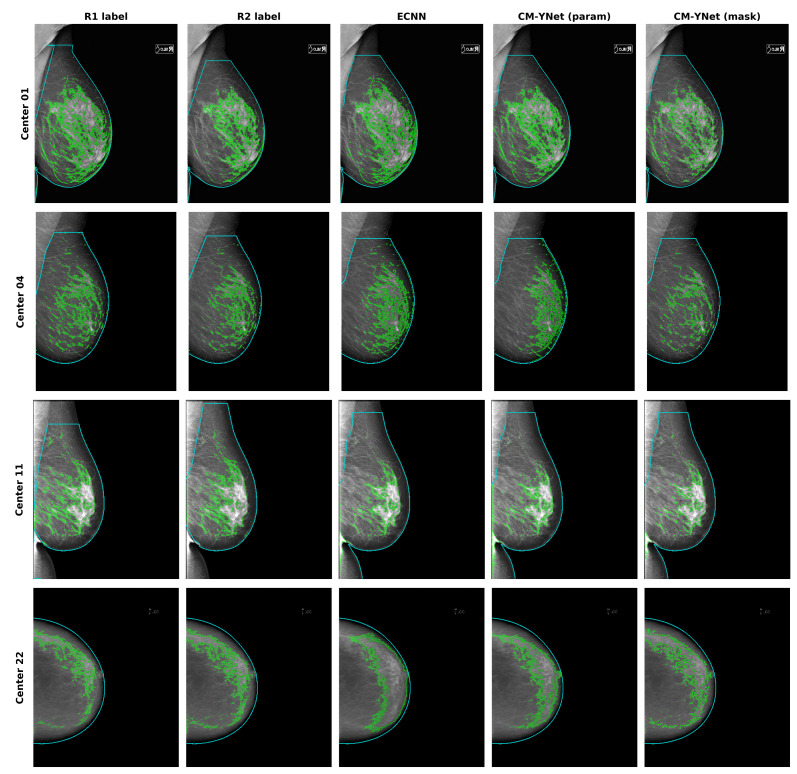
Segmentation examples for ECNN and CM-YNet compared to radiologist labels on different devices. The device of center 01 is of higher pixel-image resolution than the device of center 04. The example from center 22 is a low quality image from the IMIM dataset where the higher performance of CM-YNet (mask) is easily identified. Medio-lateral oblique mammograms were selected so the exclusion of the pectoral muscle could be seen; however, the abdomen was not excluded (center 11).

Segmentation examples for representative devices are shown in Figure 6. As can be seen in the example for center 11, the approach implemented in the current study does not manage the presence of abdomen tissue at the bottom of the image. This may have led to an additional increase in the errors reported in this study.

### 3.3. Histogram Normalization Importance

**Figure 7:**
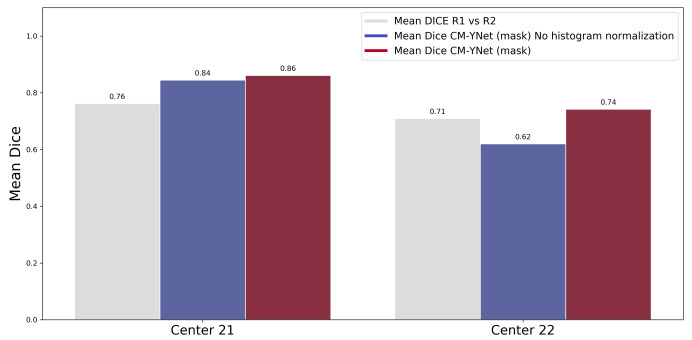
Comparison of CM-YNet (mask) segmentation with and without the preprocessing step on the IMIM dataset. The results using the proposed histogram normalization outperformed those obtained without any preprocess, especially for the low-quality images of Center 22 (p<0.05).

The previous paper [16] highlighted the importance of the normalization step described in Section 2.2.3. The results of this study further support the need for normalization of gray-level values from different sources, as can be seen in Figure 7. This plot shows the DICE scores, with and without histogram normalization, where a substantial increment in performance is seen, especially for the low-quality images of device 22 of the IMIM dataset (p<0.05).

### 3.4. Percent Density Estimation

**Figure 8:**
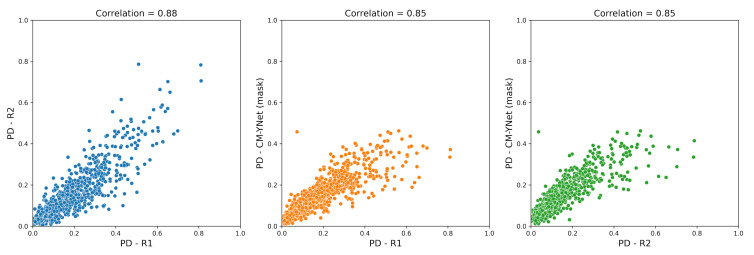
Correlations between the breast percent density (PD) calculated by our CM-YNet (mask) method and the radiologists’ annotations (R1 and R2).

Breast PD was calculated as the percentage of breast pixels segmented as dense tissue. Figure 8 shows the scatterplots for the PD estimated by CM-YNet (mask) and the PD obtained from the radiologists’ annotations. The PD estimated by our method correlated highly with both expert readers (Pearson’s coefficient, 0.85).

**Figure 9:**
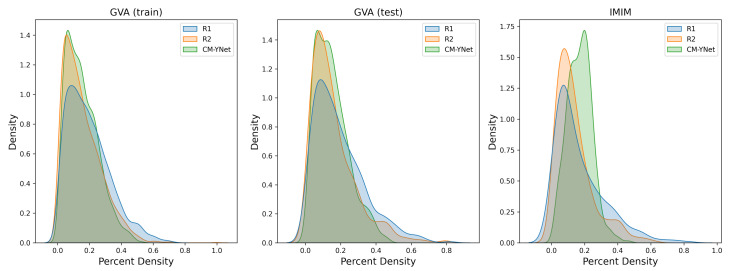
Density plots for the percent density (PD) annotated by the two experts (R1 and R2) and the mask output of the CM-YNet model.

In Table 4, we included the Dice score and the PD for each radiologist to further analyze the variability. The PD calculated by R1 was larger than R2 in 73% of the samples in the training dataset and the difference in means was statistically significant (p<0.00001). This indicates that R1 tended to over-segment in comparison to R2. Figure 9 shows the distributions for each radiologist and the mask output of the CM-YNet model.

### 3.5. INbreast Dataset

**Figure 10:**
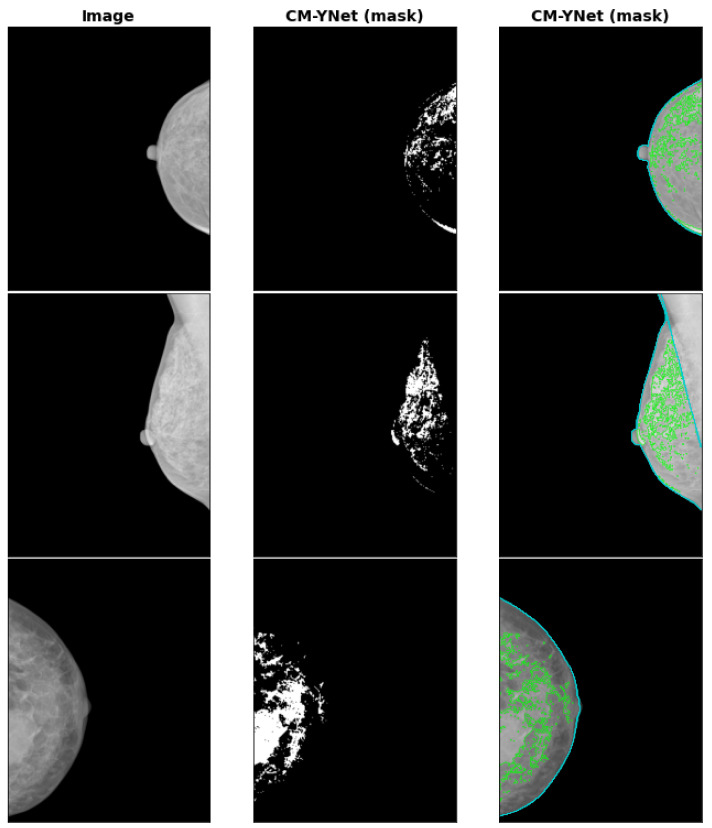
Segmentation examples for CM-YNet (mask) on the INbreast datastet.

The INbreast database [26] is a well-known publicly available dataset that includes 440 mammograms from 115 patients. It has ground-truth annotations for mass location, mass type, and breast density classification labels. However, annotations for the dense tissue mask are not available. We have estimated the dense tissue masks with our CM-YNet model—some examples are shown in Figure 10. To allow other research groups to evaluate and compare their segmentation results with the masks generated by our model, we have made the masks for all INbreast images publicly available (https://doi.org/10.34740/KAGGLE/DS/2207184 accessed on 24 July 2022).

## 4. Discussion

We have presented the CM-YNet model for joint estimation of the dense-tissue segmentation mask and the parameters used for mask reconstruction in threshold-based tools. We demonstrated that directly estimating the mask at pixel level achieved superior performance to reconstructing it using the two segmentation parameters. However, the availability of the segmentation parameters is still important to maintain compatibility with threshold-based segmentation tools in cases when the radiologist disagrees with the estimated segmentation, in which case manual modifications are required. In this regard, it is important to highlight that, for clinical applications, the outputs of any automatic method should be corroborated by a medical expert. Moreover, there is also interest in using automatic methods for longitudinal studies, where thousands of images are needed to be segmented. For this latter application, any misestimation of the dense tissue segmentation is expected to have minimal impact because of the large number of images.

We also corroborated that the inclusion of the annotation network, proposed by [17], to counteract the spatial characteristics of labeling errors by multiple human annotators is more stable and facilitates the retraining of the model with more images from different annotators.

According to [27,28], one of the important tasks for computer-aided diagnosis systems is to provide an accurate and reproducible assessment of mammographic breast density. We consider that our multi-center study performed well (DICE>0.8) regarding breast density assessment using CM-Ynet, and that it constitutes a first step in the standardization of how mammographic breast density is assessed. The automated PD scores obtained by our method had a positive relationship with the manually annotated scores, and were consistent with the correlation coefficient (0.85) reported in similar studies [29,30].

In a recent study, Saffari et al. [31] presented a fully automated framework for segmentation and classification using deep learning. The DICE score obtained for the dense-tissue segmentation was 0.88. This score was calculated over the logical-AND between two expert radiologists. Their conditional generative adversarial network (cGAN) was also trained with the logical-AND masks. Their dataset included only 115 patients (33 for testing) from the INbreast dataset [26]. This dataset size was substantially smaller than the dataset used in our multi-center study (2166 patients), which may have yielded over-optimistic results. In this regard, we have provided the estimated masks by our CM-YNet model for all INbreast images so that other research groups can compare their results with ours.

The Deep-LIBRA algorithm was recently introduced [32]. It provides estimates of the total dense tissue area and the breast PD. Deep-LIBRA was trained and validated on a multi-racial, multi-institutional dataset of 15,661 images (4437 women) and released as open-source software. One limitation of the Deep-LIBRA study is that the model was only trained with images from a single manufacturer (Hologic). We tested Deep-LIBRA with our dataset. However, the results were far from satisfactory: the breast masks were wrongly estimated, and the dense tissue mask was not estimated at all in most cases. We experienced these outcomes for all centers included in our test set, even for the Hologic ones (centers 07 and 22). The reason for this could be that all the images used to train Deep-LIBRA were raw (“for processing”) FFDM images, in contrast with the “for presentation” images available in our dataset.

The current standard practice in breast density assessment is to assign a four-class categorisation according to BI-RADS (category A: almost entirely fat; category B: scattered fibroglandular densities; category C: heterogeneously dense; and category D: extremely dense), where each category corresponds to qualitative (and not quantitative) determinations of breast density. We acknowledge that deep learning algorithms can be used for direct prediction in each BI-RADS class. However, we advocate the importance of breast density estimation for the following reasons: breast density is relevant to other tasks beyond assessment of a woman’s risk for breast cancer, such as evaluating mammographic sensitivity due to masking of tumors by dense tissue [33], or assessing the effects of aspirin use and bariatric surgery on breast parenchymal patterns [34,35].

One of the main challenges of implementing automatic segmentation tools in medical imaging is the wide variety of images due to different vendors, scanner technology, and acquisition parameters [36]. This situation is even more challenging in digital mammography, especially when we deal with “for presentation” images, as in our case. In this regard, our study covered a total of 13 centers, including different vendors. We have demonstrated that the histogram normalization implemented in our pipeline yielded good results even on low-quality images.

The main contributions of the present paper can be summarized as:

1. Improvement of the previously presented ECNN model with the CM-YNet architecture that jointly estimates the segmentation parameters, segmentation mask at the pixel level, and the confusion matrices of each expert annotator.
2. Validation of the importance of the preprocessing protocol that standardizes the histograms of breast images. This preprocessing reduces the impact of using different acquisition devices, especially when images acquired with a specific device were never seen during training.
3. The inclusion of a totally independent dataset used only for testing. This dataset allowed us to further corroborate the validity of our multi-center study showing that the generalization performance was still acceptable (DICE>0.7) even for low-quality images, such as those of Center 22.
4. The approach followed achieves higher performance than the concordance between the radiologists and also makes it easy for a radiologist to perform a fine-tuning of the results by interactively modifying the segmentation parameters using a threshold-based tool.

### Limitations and Future Research

While the CM-YNet hybrid approach improved the results of the fully parametric models, the results presented are based on comparing the DICE scores between the automatic model and the closest radiologist for each sample. This was performed due to the absence of a unique ground truth. Future work will involve including more than two expert labels in order to compare the results with fusion label methods such as majority voting or STAPLE [37].

A second limitation is the pectoral muscle exclusion algorithm. The solution adopted in the present work, although robust, could be improved by taking into account other approaches mentioned in Section 2.2.2. The use of “for presentation” mammograms instead of “raw” images may be the reason for some of the differences among the acquisition devices. It would also be interesting to check if “raw” mammograms would avoid the preprocessing step.

Finally, the estimation of the breast segmentation mask can be jointly estimated with a dense-tissue mask which would probably increase performance and decrease the segmentation time.

## 5. Conclusions

Our results show that directly estimating the dense-tissue mask at pixel level yielded a better generalization performance than predicting two threshold parameters to later reconstruct the mask, even when training samples were labeled using a threshold-based tool. Our proposed model also estimates these parameters to maintain compatibility with threshold-based segmentation tools. It was also shown that, considering each expert label as a noisy approximation of the ground truth, by jointly learning the annotator’s confusion matrices to capture each expert variability, yielded better results for each tested model.

## Figures and Tables

**Table 1:** Summary of the screening centers, their mammography devices and the number of women and mammograms per device.

Id	Center	Device	#Women	#Images
01	Castellón	FUJIFILM	191	382
02	Fuente de San Luis	FUJIFILM	190	380
04	Alcoi	IMS s.r.l./Giotto IRE ^(*)^	66	132
05	Xàtiva	FUJIFILM	159	318
07	Requena	HOLOGIC/Giotto IRE ^(*)^	28	56
10	Elda	SIEMENS/Giotto IRE ^(*)^	311	622
11	Elche	FUJIFILM	278	556
13	Orihuela	FUJIFILM	117	234
18	Denia	IMS s.r.l./Giotto IRE ^(*)^	38	76
20	Serrería	^(**)^	177	354
99	Burjassot	Senography 2000D	230	230
21	IMIM-1	FUJIFILM	98	98
22	IMIM-2	Lorad/Hologic Selenia	283	283
		**Total**	2166	3721

(*) Implies the use of a new device [Gioto IRE] since 2015. (**) The device is unknown

**Table 2:** Comparison of different models for the GVA (844 samples) and IMIM (381) datasets.

GVA Dataset				
**Model**	**R1 vs. Model**	**R2 vs. Model**	**AND vs. Model**	**Closest Radiologist**
ECNN	0.71 ± 0.23	0.72 ± 0.23	0.72 ± 0.23	0.77 ± 0.21
UNet	0.76 ± 0.15	0.76 ± 0.15	0.76 ± 0.16	0.81 ± 0.12
YNet (param.)	0.70 ± 0.21	0.70 ± 0.21	0.70 ± 0.22	0.75 ± 0.20
YNet (mask)	0.80 ± 0.14	0.78 ± 0.15	0.78 ± 0.16	0.84 ± 0.12
AND-ECNN	0.68 ± 0.21	0.64 ± 0.21	0.62 ± 0.22	0.72 ± 0.20
AND-UNet	0.76 ± 0.15	0.79 ± 0.15	0.81 ± 0.14	0.83 ± 0.12
AND-YNet (param.)	0.65 ± 0.22	0.68 ± 0.22	0.69 ± 0.23	0.72 ± 0.21
AND-YNet (mask)	0.77 ± 0.15	0.79 ± 0.15	**0.82 ± 0.14**	0.84 ± 0.12
CM-ECNN	0.71 ± 0.20	0.72 ± 0.20	0.73 ± 0.20	0.78 ± 0.18
CM-UNet	**0.80 ± 0.13**	0.80 ± 0.13	0.79 ± 0.15	**0.85 ± 0.10**
CM-YNet (param.)	0.73 ± 0.18	0.75 ± 0.17	0.77 ± 0.17	0.80 ± 0.15
CM-YNet (mask)	0.79 ± 0.13	**0.80 ± 0.13**	0.79 ± 0.15	0.84 ± 0.10
**IMIM Dataset**				
**Model**	**R1 vs. Model**	**R2 vs. Model**	**AND vs. Model**	**Closest Radiologist**
ECNN	0.58 ± 0.24	0.59 ± 0.23	0.55 ± 0.25	0.65 ± 0.23
UNet	0.67 ± 0.22	0.69 ± 0.18	0.64 ± 0.23	0.74 ± 0.18
YNet (param.)	0.60 ± 0.27	0.60 ± 0.24	0.56 ± 0.27	0.66 ± 0.25
YNet (mask)	0.69 ± 0.24	0.69 ± 0.20	0.64 ± 0.24	0.76 ± 0.20
AND-ECNN	0.58 ± 0.26	0.57 ± 0.23	0.51 ± 0.26	0.64 ± 0.24
AND-UNet	0.68 ± 0.24	0.72 ± 0.21	0.68 ± 0.25	0.76 ± 0.20
AND-YNet (param.)	0.58 ± 0.26	0.60 ± 0.24	0.57 ± 0.26	0.65 ± 0.24
AND-YNet (mask)	0.68 ± 0.24	0.71 ± 0.21	**0.68 ± 0.24**	0.76 ± 0.21
CM-ECNN	0.63 ± 0.25	0.65 ± 0.21	0.60 ± 0.25	0.71 ± 0.21
CM-UNet	**0.69 ± 0.22**	**0.72 ± 0.18**	0.66 ± 0.24	**0.77 ± 0.17**
CM-YNet (param.)	0.67 ± 0.23	0.69 ± 0.20	0.65 ± 0.23	0.74 ± 0.19
CM-YNet (mask)	0.68 ± 0.23	0.70 ± 0.19	0.64 ± 0.24	0.76 ± 0.18

DICE scores are shown as mean ± standard deviation. The last column shows the mean score between the model segmentation and the label of the closest radiologist for each sample. The highest value for each column is highlighted in bold.

**Table 3:** Comparison of DICE scores according to the different acquisition centers.

Center Id	#Images	R1 vs. R2	ECNN	CM-YNet (param.)	CM-YNet (mask)
01	96	079 ± 0.16	0.81 ± 0.16	0.75 ± 0.19	**0.81 ± 0.11**
02	96	0.79 ± 0.14	0.83 ± 0.15	0.81 ± 0.15	**0.83 ± 0.13**
04	34	0.75 ± 0.17	0.57 ± 0.23	0.74 ± 0.20	**0.83 ± 0.08** *
05	80	0.64 ± 0.17	0.84 ± 0.13	0.81 ± 0.16	**0.84 ± 0.10**
07	14	**0.88 ± 0.15**	0.85 ± 0.15	0.73 ± 0.18	0.82 ± 0.14
10	156	0.77 ± 0.16	0.68 ± 0.24	0.79 ± 0.15	**0.85 ± 0.10** *
11	140	0.82 ± 0.12	0.87 ± 0.10	0.84 ± 0.10	**0.87 ± 0.07**
13	60	0.78 ± 0.12	0.86 ± 0.12	0.82 ± 0.13	**0.86 ± 0.11**
18	20	0.74 ± 0.14	0.51 ± 0.27	0.80 ± 0.13	**0.86 ± 0.08** *
20	90	0.78 ± 0.16	0.61 ± 0.27	0.78 ± 0.16	**0.83 ± 0.12** *
99	58	0.79 ± 0.13	0.78 ± 0.20	0.83 ± 0.13	**0.89 ± 0.09** *
21	98	0.76 ± 0.14	0.80 ± 0.15	0.82 ± 0.17	**0.86 ± 0.10**
22	283	0.71 ± 0.22	0.59 ± 0.22	0.72 ± 0.19	**0.74 ± 0.19** *
Total	1225	0.76 ± 0.17	0.73 ± 0.22	0.78 ± 0.17	**0.82 ± 0.14**

Images from the centers 21 and 22 are from the independent IMIM dataset in which id 22 corresponds to images acquired with old devices. The highest value for each row is highlighted in bold. * The difference between CM-YNet (mask) and ECNN is statistically significant (*p* < 0.001).

**Table 4:** Percent density (PD) and generalized energy distance (GED) for CM-YNet (mask).

Dataset	#Images	R1 vs. R2	PD-R1	PD-R2	%(R1 > R2)	GED
GVA(train)	2496	0.76 ± 0.17	0.19 ± 0.13 *	0.16 ± 0.12	73.31	3.13 ± 0.44
GVA(test)	844	0.77 ± 0.15	0.19 ± 0.14 *	0.16 ± 0.13	68.36	3.16 ± 0.40
IMIM	381	0.72 ± 0.20	0.17 ± 0.15	0.14 ± 0.11	62.2	2.80 ± 0.56

The DICE score between R1 and R2, and the percent density (PD) for each annotator are shown as mean ± standard deviation. The percentage of samples in which PD-R1 was larger than PD-R2 is shown in the column %(R1 > R2). The last column shows the generalized energy distance (GED) between the annotator’s labels and the estimated segmentation for each annotator. * The difference between the percent density (PD) obtained by R1 and R2 was statistically significant (*p* < 0.00001)

## Data Availability

The masks generated by our CM-YNet model for all INbreast images as described in Section 3.5 are available at: https://www.kaggle.com/datasets/itiresearch/inbreastdensetissue (accessed on 24 July 2022).
